# Cognitive Impairment Involving Social Cognition in SPG4 Hereditary Spastic Paraplegia

**DOI:** 10.1155/2016/6423461

**Published:** 2016-09-04

**Authors:** Ludivine Chamard, Sabrina Ferreira, Alexa Pijoff, Manon Silvestre, Eric Berger, Eloi Magnin

**Affiliations:** ^1^University Hospital of Besançon, CH Jean Minjoz, Besançon, France; ^2^Regional Hospital of Dole, CH Louis Pasteur, Dole, France

## Abstract

*Objectives*. To describe cognitive assessment including social cognition in SPG4 patients.* Methods*. We reported a series of nine patients with SPG4 mutation with an extensive neuropsychological examination including social cognition assessment.* Results*. None of our patients presented with mental retardation or dementia. All presented with mild cognitive impairment with a high frequency of attention deficit (100%), executive disorders (89%), and social cognition impairment (78%). An asymptomatic patient for motor skills presented with the same cognitive profile. No correlation was found in this small sample between cognitive impairment and motor impairment, age at disease onset, or disease duration.* Conclusions*. SPG4 phenotypes share some cognitive features of frontotemporal lobar degeneration and amyotrophic lateral sclerosis. Cognitive disorders including executive disorders and social cognition impairment are frequent in SPG4 patients and might sometimes occur before motor disorders. Therefore, cognitive functions including social cognition should be systematically assessed in order to improve the clinical management of this population.

## 1. Introduction

Hereditary spastic paraplegia (HSP) is a heterogeneous group of complex inherited disorders in which the main clinical feature is a motoneuron disease with progressive spasticity and weakness of the lower limbs. Autosomal dominant transmission is a frequent feature in HSP. The most frequent autosomal dominant HSP is due to mutations or deletions of the SPAST gene encoding the microtubule-severing protein spastin which is located on the chromosome 2p (SPG4 locus) [1].

Some authors report subtle cognitive decline [2] while others report dementia [3] or mental deficiency [4] in SPG4 HSP patients. Most of these studies used a global cognitive efficiency test such as mini-mental state examination (MMSE), Mattis dementia rating scale, short test of mental status (STMS), Cambridge cognitive examination (CAMCOG), and Wechsler adult intelligence scale (WAIS) [4, 5]. However, some studies used more exhaustive neuropsychological assessments [6, 7]. Executive functions and verbal episodic, face recognition, and short term and working memories are frequently impaired. These cognitive disorders generally occur during aging in the sixth decade [6–8]. As cognitive performances are compared with those of age-matched control groups, the cognitive impairment observed in older SPG4 mutation patients suggests a specific neurodegenerative process in addition to cognitive modification that occurred during normal aging.

The main pathological finding in HSP is a motoneuron disease with axonal degeneration of the terminal portions of the corticospinal tract of the spinal cord [3, 9]. In patients with dementia that presented with a deletion of exon 17 of the SPAST gene, the neuropathological examination showed widespread ubiquitin positivity within the neocortex and white matter and rare tau-positive lesions in the frontal, temporal regions [10]. In patients with missense mutation in exon 10, tau-positive lesions are also reported in limbic regions [11].

Executive and social cognition impairments are reported in amyotrophic lateral sclerosis patients [12, 13], another motoneuron disease. The neuropathological findings (i.e., ubiquitin-positive lesions and tau-positive frontotemporal and limbic lesions) and neuropsychological features (including frequent executive disorders) suggest some similarities between SPG4 HSP and frontotemporal dementia. Therefore, as social cognition disorders are frequent in frontotemporal dementia [14] and in another motoneuron disease such as amyotrophic lateral sclerosis [12, 13], we hypothesize that social cognition might be impaired in SPG4 HSP patients.

The aim of our study was to describe the neuropsychological profile (including social cognition) of a series of SPG4 participants.

## 2. Methods

**Figure 1:**
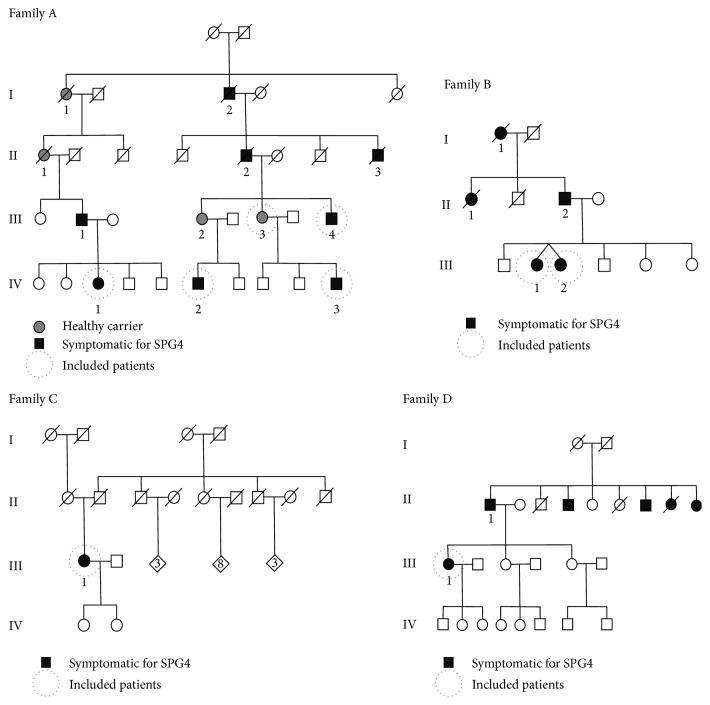
Pedigrees of the four families.

Eight affected participants and an asymptomatic one (all carriers of an identified mutation in the SPAST gene) from 4 families were included (6 females and 3 males) (Figure 1). Family A (5 participants), family B (2 patients), family C (1 patient), and family D (1 patient) presented with c.1675G>C (p.Gly559Arg), c.1413+2T>A intronic, c.1378C>T (p.Arg460Cys), and c.1536+1G>T (splicing abnormality) heterozygote mutations, respectively (Figure 1). Written informed consent was obtained from all patients. Systematic neurological and extensive neuropsychological assessments were performed. The spinocerebellar degeneration functional score (SDFS) was systematically used to evaluate the disability stage from 0 to 7 (stage 0 corresponds to a patient with no functional handicap and no neurological symptom; stage 1 corresponds to a patient with no functional handicap but neurological symptoms observed during medical examination; stage 2 corresponds to a patient with mild disabilities presenting a preserved ability to run and an unlimited walking perimeter; stage 3 corresponds to patients with moderate disabilities presenting running difficulties and limited walking perimeter; stage 4 corresponds to a patient with severe disabilities requiring one stick to walk; stage 5 corresponds to a patient presenting severe disabilities requiring two sticks to walk; stage 6 corresponds to patient presenting no more walking abilities and requiring wheelchair; stage 7 corresponds to a patient restricted to bed). The RAPID neuropsychological battery [15] (including mini-mental state examination (MMSE) evaluating overall global cognitive efficiency, the 16-item Free and Cued Recall Test assessing verbal episodic memory with 4 lists of 4 words (16 items) to read and memorize permitting evaluating encoding (immediate recall), retrieval (free recall), and storage using categorical cueing (sum of Free and Cued Recall), the Trail Making Test (TMT) part A and Crossing Off Test assessing information processing speed, the TMT part B, and the Isaacs Set Test assessing executive functions; the TMT and geometric figure copy assessing visuospatial abilities and the 30-item Picture Naming Test assessing visuoperceptive and language abilities) was performed. In addition, all participants performed the Beehive Visual Memory Test to assess their visuospatial episodic memory by memorizing 10 blackened cases in a checkerboard of 49 cases, the forward and backward digit and visuospatial spans to assess verbal-auditory and visuospatial short term and working memory, gestural assessment to screen apraxia, Test of Attentional Performance (TAP) to assess alertness, flexibility, divided attention, and incompatibility, Wisconsin Card Sorting Test to assess executive function (such as flexibility, inhibition, and abstract concept) and the mini-social cognition and emotional assessment (SEA) to assess recognition of facial emotions and theory of mind (faux pas test) [16, 17]. The Instrumental Activities of Daily Living (IADL) scale was performed to evaluate functional autonomy [18]. All neuropsychological tools are normed and validated to be applied at an individual level with age, gender (when available), and education-adjusted cut-off scores (percentiles or standard deviations). As the sample was quite small, only Spearman's correlation was performed between the number of impaired cognitive functions and other clinical and demographical numerical data (age, age at onset, disease duration, level of education, MMSE, and SDSF).

## 3. Results

The mean age at examination was 52 (±8.7); the mean period of education was 8.9 years (±2.6). Six participants were right-handed and 3 were ambidextrous. The disease duration of symptomatic patients was 16.1 years (±11.7). The mean age of symptom onset was 33.9 years (±12.2; [12–45]). The mean of the SDFS was 2.9 (±1.5). Two participants (family B, patients III1 and III2) were monozygotic twins.

The neuropsychological assessment (Table 1) showed a mean score of the MMSE at 26.4 (±3.3). No impairment was reported in the IADL assessment. However, 2 patients (FBIII1 and FBIII2) presenting a ceiling effect on the MMSE (i.e., 30/30) had impairments, respectively, in 3 and 4 different cognitive domains. All participants had attention deficit; 89% (8/9 patients) had executive disorders. One participant had apraxia. One patient had semantic memory impairment. No aphasia or agnosia was observed. 78% (7/9 patients) had social cognition impairment (total mean score 21.4 (±3.8)/30) with a systematic deficit in the recognition of facial emotions (mean subscore 10.2 (±2.5)/15) and 44% (4/9 patients) had also a deficit in the faux pas test (mean subscore 11.2 (±1.7)/15). 33% (3/9 patients) presented with slowness of one hand movement while just one (FAIV3) had spastic paresis of an arm. No mental retardation or dementia was observed.

The monozygotic twins (family B, patients III1 and III2) had different clinical and neuropsychological profiles. One (FBIII1) had ambidexterity while the other (FBIII2) was right-handed. The age at symptom onset was 38 for the first and 42 for the second. Both presented with social cognition impairment, executive function disorders, and dynamic apraxia and movement slowness of the left hand. Patient FBIII2 presented, in addition, with semantic memory disorders.

No correlation was found between the number of impaired cognitive domains and clinical and demographical numeric data (age, age at onset, disease duration, level of education, MMSE, and SDSF).

## 4. Discussion

No dementia, intellectual disability, language disorders, or information processing speed impairment was observed in our series whereas those phenotypes are frequently reported in the literature [3–7]. In our series, only one patient presented with a deficit of semantic memory and one patient presented with apraxia while it is frequently reported in other studies [2, 5–7, 10]. In addition, the high frequency of attention deficit and executive impairment (resp., 100% and 89%) is more substantial than expected from the literature [2, 4–7]. This difference might be explained by the small sample with only 4 different mutations and 5 patients (family A) out of 9 carrying the same mutation in our series, while clinical heterogeneity, due to the genetic heterogeneity with mutations in more than 70 loci, is frequently reported in SPG4 population. The small number of SPG4 patients and the small number of different families of our series are a limitation of our study. Another limitation is the absence of a similar neuropsychological assessment in an age, gender, and education-level matched control group of healthy participants that might also explain the difference observed in the neuropsychological profile of our series in comparison with previous results reported in the literature. However, all neuropsychological tools used to evaluate cognitive impairment in our study were normed and validated with age, gender (when available), and education-adjusted cut-off scores [15–17]. Using normative cut-offs reflected the standard procedure in daily clinical practice of neurology clinics to evaluate patients at an individual level. Therefore, future studies focused on cognition in SPG4 patients with larger samples and larger number of SPG4 mutations and control groups are needed to confirm our results.

All our patients presented with cognitive impairment fulfilling multidomain nonamnesic mild cognitive impairment (MCI) criteria [19, 20], while cognitive impairment is usually reported as frequent but inconstant [7]. As the mean MMSE score was 26.6 and 2 patients with a score of 30 presented nevertheless with multiple cognitive domain impairments, the MMSE seems to be insufficiently sensitive to screen cognitive impairment in this population. MCI and executive impairment frequency might be underestimated in the literature by the use of the global cognitive efficiency test instead of extensive neuropsychological assessments that specifically evaluate attention, executive function, and social cognition in the population of SPG4 patients. Montreal Cognitive Assessment (MoCA) and Frontal Assessment Battery (FAB) are reported as useful tools to screen global cognitive and executive disorders in amyotrophic lateral sclerosis [21] and therefore could be interesting in cognitive screening of SPG4.

The fact that cognitive disorders occurred during adulthood confirms a neurodegenerative process occurring in SPG4 mutation patients [8]. However, no correlation was found in our series between cognitive disorders and age at disease onset or disease duration. As it was a small sample, this result should be interpreted with caution and confirmed in a larger series of SPG4 carriers with and without motor symptoms. The clinical and neuropsychological differences observed between the twins are interesting. FBIII2, the twin that presented the youngest disease onset, the longer disease duration, and the most important motor impairment, presented also more cognitive domain deficits than her sister. To our knowledge, this is the first report of SPG4 twins in the literature. As the twins share the same DNA and the same education, these results suggest epigenetic phenomena that modulate SPG4 mutation phenotype.

In addition, we observed slow movement of one hand in 62.5% of SPG4 persons, while only 11% had spasticity in the upper limbs and none had motor impairment. This suggests that presymptomatic early upper limb motor disorders could be observed with timed examinations, like the Test of Attentional Performance.

For the first time, social cognition was assessed in SPG4 mutation. Executive and social cognition disorders were frequently observed in our series of SPG4 patients. The deficits observed are suggestive of a cortical disorder instead of a subcortical one. Those cognitive profiles are similar to those observed in early predemential frontotemporal neurodegenerative disorders [14]. Those results are concordant with neuropathological studies that showed neocortical lesions including limbic regions [11] and neuroimaging ones that showed a left frontotemporal hypoperfusion in a SPG4 HSP population [6].

Social cognition impairment is described in amyotrophic lateral sclerosis patients without frontotemporal lobar dementia and mirror neurons are supposed to play a critical role in this function [12, 13]. In addition to the widespread ubiquitin positivity deposit and tau-positive frontotemporal and limbic lesions, motor neuron disorders, modifying mirror neurons networks, might sometimes directly participate in the social cognition impairment in SPG4. However, 2 patients (FAIV1 and FDIII1) that presented with motor disorders did not present social cognition impairment: this suggests that other mechanisms might compensate for the motor neuron and mirror neuron dysfunctions. Nevertheless, the asymptomatic FAIII3 participant presented with the same neuropsychological profile compared with symptomatic participants with social cognition disorders including both recognition of facial emotions and theory of mind impairments, attention deficit, and executive disorders. Therefore, neuropsychological profile of our asymptomatic FAIII3 participant raises the question of cognitive disorders as potential prodromal independent symptoms of SPG4 mutation. In particular, social cognition impairment might suggest an early dysfunction of the frontal lobe or motor neurons/mirror neurons complex in presymptomatic SPG4 patients. A larger group of “asymptomatic” patients with SPG4 mutation would be interesting for evaluating this early symptom in a future study. If this cognitive profile is confirmed, criteria for premotor symptoms of SPG4 should be included, and a neuropsychological assessment should be systematically performed to suggest a SPAST mutation. Social cognition impairment could have an impact on social and professional insertion and should therefore be considered as an important symptom to screen in SPG4 patients.

In conclusion, this study suggests that all SPG4 participants, including “asymptomatic” carriers (for the motor function), have cognitive impairment. Social cognition impairment, reported for the first time, and executive disorders might be frequent in this pathology. A neurodegenerative process may be involved in SPG4 relative disorders that share some features of frontotemporal lobar degeneration and amyotrophic lateral sclerosis. Therefore, these functions should be systematically assessed by an extensive neuropsychological assessment to improve the clinical management of this population.

## Figures and Tables

**Table 1:** Neuropsychological assessment of SPG4 participants.

Patient	Age	Sex	Level of education (years)	MMSE	Episodic memory	Semantic memory	Executive function	Attention	Language	Gnostic function	Praxis function	Social cognition	Number of altered domains	Age at disease onset (years)	Disease duration (years)	SDSF
FAIII3	68	f	5	27			X	X				X	3	NA	NA	0
FAIII4	64	m	4	19			X	X				X	3	45	19	4
FAIV1	45	f	9	28				X					1	12	33	5
FAIV2	50	m	10	25			X	X				X	3	44	6	2
FAIV3	43	m	10	27			X	X			X	X	4	30	13	3
FBIII1	50	f	11	30			X	X				X	3	42	8	3
FBIII2	50	f	11	30		X	X	X				X	4	38	12	4
FCIII1	54	f	9	26			X	X				X	3	20	34	3
FDIII1	44	f	11	26			X	X					2	40	4	2

SDSF: spinocerebellar degeneration functional score, NA: not applicable, f: female, m: male, and MMSE: mini-mental state examination. A cross means an impairment of the cognitive function.
